# Role of natriuretic peptides in the diagnosis and treatment of patients with carcinoid heart disease

**DOI:** 10.1038/sj.bjc.6601816

**Published:** 2004-04-20

**Authors:** J M Zuetenhorst, C M Korse, J M G Bonfrer, R H Bakker, B G Taal

**Affiliations:** 1 Department of Gastroenterology, Netherlands Cancer Institute/Antoni van Leeuwenhoek Hospital, Amsterdam, The Netherlands; 2 Department of Clinical Chemistry, Netherlands Cancer Institute/Antoni van Leeuwenhoek Hospital, Amsterdam, The Netherlands; 3 Department of Cardiology, Slotervaart Hospital, Amsterdam, The Netherlands

**Keywords:** carcinoid heart disease, urinary 5-HIAA, ANP, BNP, Chromogranin A

## Abstract

Carcinoid heart disease (CHD) occurs in 20–70% of the patients with metastatic well-differentiated neuroendocrine tumours (NET). We evaluated whether natriuretic peptides (ANP or NT-proBNP) are useful in early detection of CHD. Blood samples from 32 patients with NET were compared with cardiac ultrasound follow-up. CHD was defined as thickening of the tricuspid valve in the presence of grade III–IV/IV tricuspid valve regurgitation. CHD was found in nine out of 32 patients (28%), all with symptoms of the carcinoid syndrome compared to 65% in the 23 patients without CHD (*P*=0.04). Median levels of NT-proBNP and 5-HIAA were significantly higher in patients with CHD (894 ng l^−1^ and 815 *μ*mol 24 h^−1^) compared to those without (89 and 206 ng l^−1^, *P*<0.001 and *P*=0.007). No significant differences were detected in ANP levels (*P*=0.11). Dilatation of the right atrium and ventricle as well as thickening of the tricuspid valve and degree of regurgitation were statistically significant correlated with NT-proBNP levels. The accuracy of NT-proBNP in the diagnosis of CHD was higher than that of ANP. A significantly better survival was observed in case of normal NT-proBNP values. In conclusion, NT-proBNP is helpful as a simple marker in the diagnosis of CHD. Survival is better in patients with normal levels of NT-proBNP.

In 1981, De Bold *et al* (1981) first described the endocrine function of the heart with natriuretic and diuretic effects. These hormonal activities were later linked to peptides such as atrial natriuretic peptide (ANP) and brain natriuretic peptide (BNP). The peptides are produced primarily within the atria and ventricles of the heart and are released into the circulation in response to increased wall tension, thus reflecting increased intravascular volume. Both ANP and BNP are produced as propolypeptides (pro-ANP and pro-BNP) and are cleaved after excretion into the biological active peptides (ANP and BNP) and an inactive N-terminal fragments (NT-proANP and NT-proBNP). Both active and inactive peptides can be isolated from the blood, but the stability of the prohormones and NT-terminal fragments is much higher compared to the activated form. After activation, natriuresis starts and a decrease in blood pressure occurs as a result of shifting intravascular fluid into the extravascular compartment and suppression of the rennin–angiotensin–aldosteron axis.

Well-differentiated neuroendocrine tumours (NET) with liver metastases can give symptoms of the characteristic carcinoid syndrome with diarrhoea and flushes caused by the overproduction of serotonin. Carcinoid heart disease (CHD) is a well-known complication of long-lasting exposure to high levels of serotonin (Tornebrandt *et al*, 1986; Lundin *et al*, 1988; Robiolio *et al*, 1995; Westberg *et al*, 2001; Zuetenhorst *et al*, 2003). Many carcinoid patients die from cardiac causes (Ross and Roberts, 1985) and the detection of CHD in an early stage is important to adjust therapy and hence improve prognosis.

Large studies in the general population or in noncardiac patients showed that measuring natriuretic peptides might be an effective screening method for left-ventricular systolic dysfunction (McDonagh *et al*, 1998; Luchner *et al*, 2000; Bay *et al*, 2003). In patients with the suspicion of heart failure several other studies showed natriuretic peptides to be useful indicators for the detection of heart failure (Lerman *et al*, 1993; Davidson *et al*, 1996; Cowie *et al*, 1997; Hammerer-Lercher *et al*, 2001; Maisel *et al*, 2002,^2003^). In the follow-up of patients with an acute cardiac event levels of natriuretic peptides were proved to be of prognostic value for outcome (Hall *et al*, 1994; Omland *et al*, 1996; de Lemos *et al*, 2001; Koglin *et al*, 2001; Richards *et al*, 2003).

Studies about the role of natriuretic peptides in patients with NET are rare. In a report of Lundin *et al* (1989) ultrasound studies were performed in 50 patients and combined with blood atrial natriuretic peptide concentrations. In patients with clinical findings of right ventricular failure significantly higher levels of ANP were found. However, no studies have been performed to determine the diagnostic value of BNP in patients with CHD.

In this study, we investigated the relationship between CHD and the blood levels of NT-proBNP and ANP as markers for cardiac (dys)function. We also examined survival of patients with and without elevated levels of these natriuretic peptides in order to evaluate the prognostic value of these hormones.

## PATIENTS AND METHODS

Cardiac ultrasound studies were performed in 32 consecutive patients with NET (18 women and 14 men) who visited the outpatient department of the Netherlands Cancer Institute/Antoni van Leeuwenhoek Hospital in 1999 and 2000 for follow-up. The mean age was 61 years (range 34–77 years). The median interval between the diagnosis of metastatic NET and the cardiac investigation plus laboratory testing was 22 months (range 2–121 months).

### Cardiac ultrasound imaging

Two-dimensional echocardiography with continuous wave Doppler and colour flow Doppler studies were performed using standard techniques (Hewlett-Packard Sonos 5500 with 2.0/2.5 MHz probes). Echocardiographic parameters analysed were: valve morphology (normal or thickened), valve mobility (normal, mildly-, moderately-, severely diminished, fixed), valve regurgitation (none, I–IV/IV), valvular stenosis and atrial/ventricular dimensions. The criteria for CHD in our study was: a thickened tricuspid valve with additional III/IV or IV/IV tricuspid valve regurgitation (Zuetenhorst *et al*, 2003).

### Laboratory techniques

Urinary 5-HIAA excretion and levels of NT-proBNP and ANP were determined at the same time as the cardiac investigation. A routine of 24 h urine samples were collected and qualitatively evaluated for 5-HIAA and analysed by reversed-phase HPLC (normal <40 *μ*mol 24 h^−1^). A fluorescence detector was used for detection and quantification (Stroomer *et al*, 1990).

Serum levels of NT-proBNP were determined in serum by an electrochemiluminescence immunoassay used on the Modular Analytics E170 (Roche Diagnostics, Mannheim, Germany). Normal levels of NT-proBNP are affected by age (under or above 50 years) and gender. According to instructions of the manufacturer, in patients above 50 years the cutoff value for healthy women is 155 ng l^−1^ and for men 222 ng l^−1^. For practical reasons, we decided to use a cutoff value of 200 ng l^−1^, because all our patients except two were aged above 50 years. Atrial natriuretic peptide (ANP) was measured in plasma samples using an IRMA assay manufactured by CIS bio international, Gif-sur-Yvette, France (normal value <43 ng l^−1^). Determination of NT-proBNP was performed in all patients, ANP in 27 out of 32 patients (eight with CHD and 19 without CHD).

Chromogranin A levels were determined in serum using a solid-phase two site immunoradiometric assay (normal <120 *μ*g l^−1^). Two monoclonal antibodies were prepared against sterically remote sites on the CgA molecule. The first one is coated on the tube and the second one, radiolabelled with iodine 125 is used as a tracer (CIS bio international, Gif-sur-Yvette, France) (Degorce *et al*, 1999).

### Histology

Histology was classified into low-grade (<10 mitoses per 2 mm^2^ without necrosis) and high-grade neuroendocrine tumours (>10 mitoses per 2 mm^2^ and/or necrosis) according to the revised classification described by Capella *et al* (1995).

### Statistics

Comparisons between the CHD and the non-CHD group were made by the Mann–Whitney test or the Kruskal–Wallis test in case of a continuous variable. Dichotomous variables were tested by means of the Fisher's exact test.

## RESULTS

Tricuspid valvular lesions combined with regurgitation as described in our criteria for CHD were found in nine out of 32 patients (28%). Additionally, severe dilatation of the right atrium was present in almost all (eight out of nine) patients with CHD, while severe dilatation of the right ventricle was found in three non-CHD patients (Table 1

**Table 1:** Echocardiographic findings in carcinoid patients (*n*=32) according to the presence of heart disease

	**Without carcinoid heart disease (*n*=23)**	**Carcinoid heart disease (*n*=9)^a^**
*Right atrium*		
Normal	21 (91%)	0 (0%)
Mildly dilated	2 (9%)	1 (11%)
Severely dilated	0 (0%)	8 (89%)
		
*Right ventricle*		
Normal	22 (96%)	1 (11%)
Mildly dilated	1 (4%)	5 (56%)
Severely dilated	0 (0%)	3 (33%)
		
*Tricuspid valve*		
Thickened	2 (9%)	9 (100%)
Normal	21 (91%)	0 (0%)
		
*Tricuspid regurgitation*		
None	8 (35%)	0 (0%)
I/IV	8 (35%)	0 (0%)
II/IV	7 (30%)	0 (0%)
III/IV	0 (0%)	3 (33%)
IV/IV	0 (0%)	6 (67%)

a Defined as: thickening of the tricuspid valve with additional III/IV or IV/IV tricuspid valve regurgitation.

).

In 29 out of 32 patients (91%) liver metastases were present. In six patients urinary 5-HIAA excretion was normal, while it was elevated in 26 patients (median 369 *μ*mol 24 h^−1^, range 54-1185 *μ*mol 24 h^−1^). Patients with CHD had a significant longer history of liver metastases compared to those without CHD (median duration 40 and 14 months, respectively, *P*=0.02) (Table 2

**Table 2:** Clinical characteristics in carcinoid patients according to the presence of heart disease

	**Total group (*n*=32)**	**Without carcinoid heart disease (*n*=23)**	**Carcinoid heart disease^a^** **(*n*=9)**	***P*-value**
*Age at cardiac ultrasound (years)*				
Mean (range)	61 (34–77)	61 (34–76)	65 (51–77)	0.81
				
*Sex*				
Male	14 (44%)	9 (39%)	5 (55%)	0.41
Female	18 (56%)	14 (61%)	4 (45%)	
				
*Duration of carcinoid disease at echocardiogram (months)*				
Median (range)	22 (2–121)	20 (2–121)	40 (9–96)	0.08
				
*Liver metastases*	29 (91%)	21 (88%)	9 (100%)	0.36
				
*Duration of liver metastases (months)*				
Median (range)	31 (2–96)	14 (2–84)	40 (9–96)	0.02
				
*Symptoms of carcinoid syndrome*				
Yes	24 (76%)	15 (65%)	9 (100%)	0.04
No	8 (24%)	8 (35%)	0 (0%)	
				
*Primary tumour*				
Foregut	2 (6%)	2 (9%)	0 (0%)	0.16
Midgut	15 (47%)	11(48%)	4 (45%)	
Hindgut	1 (3%)	0 (0%)	1 (10%)	
Unknown	14 (44%)	10 (43%)	4 (45%)	
				
*Pathology*				
Low-grade NET^b^	24 (76%)	16 (69%)	8 (90%)	0.33
High-grade NET	5 (16%)	5 (22%)	0 (0%)	
Cytological function	3 (5%)	2 (9%)	1 (10%)	
				
*NT-proBNP (normal <200 ng l^−1^)*				
Median (range)	155 (23–4432)	89 (23–1449)	894 (328–4432)	<0.001
				
*ANP (normal <43 ng l^−1^)*				
Median (range)	26 (10–89)	25 (10–57)	41 (12–89)	0.11
				
*5-HIAA (normal <40 μmol (24 h)^−1^)*				
Median (range)	292 (19–1185)	206 (19–1116)	815 (87–1185)	0.007
				
*CgA (normal <120 μg l^−1^)*				
Median (range)	777 (24–22282)	684 (24–9115)	1958 (506–22282)	0.05

a Defined as: thickening of the tricuspid valve with additional III/IV or IV/IV tricuspid valve regurgitation.

b NET=neuroendocrine tumour.

). All CHD patients suffered from the carcinoid syndrome (flushes, diarrhoea or wheezing) compared to 65% of the non-CHD patients (*P*=0.04). No significant differences were seen between the CHD and non-CHD group in respect to age, gender, presence of liver metastases (Table 2).

During sample collection a total of 20 out of 32 patients were treated with somatostatin analoga. Pharmacological doses of meta-iodobenzylguanidine (MIBG) were administered in 18 patients, two of them during sample collection. Nine patients received a combination with radioactive labelled MIBG (Taal *et al*, 1996,^2000^), all but one at least 3 months before blood collection. In all, 14 patients were treated with interferon, none of them during collection time. There were no significant differences in these treatment modalities between CHD and non-CHD patients.

^111^In-pentetreotide scintigraphy was available in 31 out of 32 patients. A positive scan was found in 26 out of 32 (81%) patients and five patients had a negative scan. In four of these five patients, the primary tumour was located in the midgut and in one patient in the foregut.

**Figure 1:**
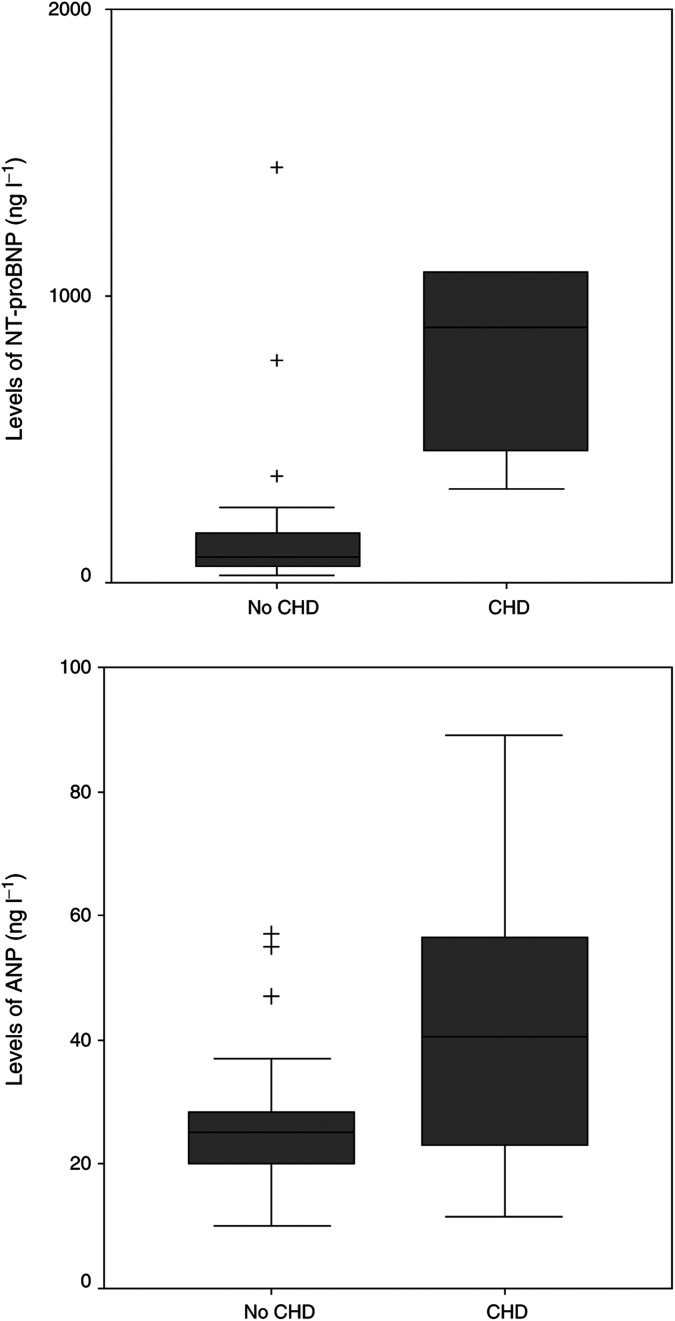
The median NT-proBNP serum level is significantly higher in patients with CHD compared to those without. The difference in ANP levels is not significant. Boxes are median and interquartiles range, whiskers show ranges excluding outliers. Values beyond the lines are considered outliers (+).

**Figure 2:**
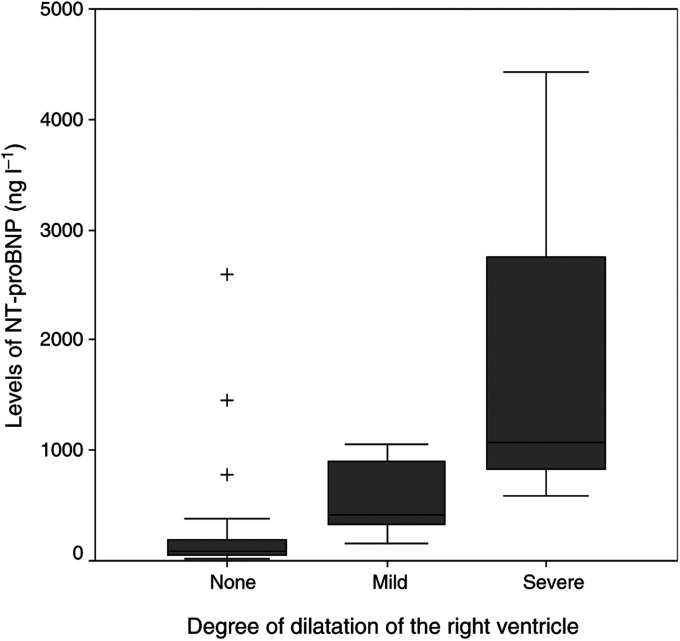
The median NT-proBNP serum level is significantly correlated with the degree of dilatation of the right ventricle. Boxes are median and interquartiles range, whiskers show ranges. Values beyond the lines are considered outliers (+).

Significantly higher median levels of NT-proBNP and urinary 5-HIAA were found in the patients with CHD (894 ng l^−1^ and 815 *μ*mol 24 h^−1^, respectively) compared to those without CHD (89 and 206 ng l^−1^; *P*<0.001 and *P*=0.007, respectively) (Figure 1

and Table 2). Median CgA levels were also found to be significantly higher in patients with CHD (1958 *μ*g l^−1^) compared with the non-CHD group (684 *μ*g l^−1^, *P*=0.05). No significant differences were detected in the levels of ANP between both groups (*P*=0.11) (Figure 1). Although levels of NT-proBNP are affected by age (under or above 50 years) and gender, we applied a fixed cut-off value of 200 ng l^−1^ because all our patients except two had an age above 50 years. In two patients (both women) with an age under 50 years (34 and 47, respectively) the NT-proBNP levels were beneath 60 ng l^−1^. The advised cutoff value for this group is 155 ng l^−1^, using our cutoff point of 200 ng l^−1^ did not make any difference in our study population. For ANP, no differences in levels between men and women are described and a correlation with age is weaker than described in BNP (Clerico *et al*, 2002). The serum concentration of NT-proBNP was elevated in all patients with CHD. ANP levels were elevated in four out of seven CHD patients. Elevated levels of NT-proBNP in patients with reported normal echocardiographic findings were found in four out of 23 patients (median 575 ng l^−1^, range 266–1449). In three of these patients thickening of the tricuspid valve with grade II/IV tricuspid regurgitation was already present. During follow-up 1 year later, one of these patients met our criteria for CHD. The other two died before a new echocardiography could be performed. The fourth patient suffered from dilatation of the right atrium after a myocardial infarction. NT-proBNP was elevated in all patients with severe dilatation of either right atrium or ventricle and the level of NT-proBNP was correlated with the degree of dilatation (*P*=0.002 and 0.005, respectively) (Figure 2

) (Table 3

**Table 3:** Levels of NT-proBNP and ANP according to the echocardiographic findings

	**NT-proBNP level (ng l^−1^) (normal <200 ng l^−1^)**	**ANP (ng l^−1^) (normal <43 ng l^−1^)**
*Carcinoid heart disease*		
Median (range)		
Absent (*n*=23)	89 (23–1449)	25 (10–57)
Present (*n*=9)	894 (328–4432)	41 (12–89)
*P*-value	*P*<0.001	*P*=0.11
		
*Right atrium dilatation*		
Median (range)		
None (*n*=21)	89 (23–1449)	26 (10–57)
Mildly dilated (*n*=3)	195 (62–2587)	25 (20–30)
Severely dilated (*n*=8)	738 (328–4432)	48 (12–89)
*P*-value	*P*=0.002	*P*=0.36
		
*Right ventricle dilatation*		
Median (range)		
None (*n*=23)	84 (23–2587)	25 (10–55)
Mildly dilated (*n*=6)	407 (153–1058)	49 (12–62)
Severely dilated (*n*=3)	1081 (581–4432)	52 (16–89)
*P*-value	*P*=0.005	*P*=0.13
		
*Tricuspid valve morphology*		
Median (range)		
Normal (*n*=21)	84 (23–372)	24 (10–57)
Thickened (*n*=11)	894 (328–4432)	48 (12–89)
*P*-value	*P*<0.001	*P*=0.39
		
*Tricuspid valve regurgitation*		
Median (range)		
None (*n*=8)	50 (23–1349)	18 (10–28)
Mild (I/IV & II/IV) (*n*=15)	153 (52–1449)	26 (20–57)
Severe (III/IV &IV/IV) (*n*=9)	894 (328–4432)	41 (12–89)
*P*-value	*P*=0.007	*P*=0.13

). Elevated NT-proBNP levels were found in four out of 21 patients with normal dimensions of the right atrium (range 266–1449 ng l^−1^) and in five out of 23 patients with normal right ventricle dimension (range 266–2587 ng l^−1^). No significant correlation was detected between the median levels of ANP and the existence of atrial or ventricle dilatation (Table 3). Median NT-proBNP levels were higher in patients with pathological thickening of the tricuspid valve (894 ng l^−1^) compared to those with a normal aspect of the tricuspid valve (84 ng l^−1^, *P*<0.001). Elevated levels of NT-proBNP were present in all patients with severe tricuspid valve regurgitation and significantly correlated with the degree of regurgitation (*P*=0.007). Such significant findings were not found in the levels of ANP (Table 3).

**Figure 3:**
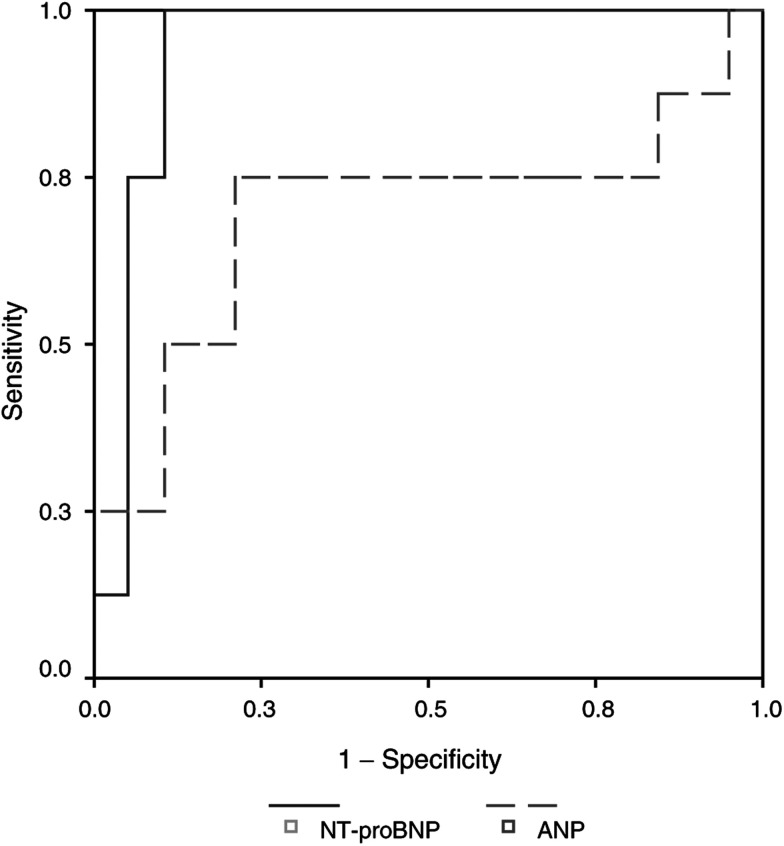
The ROC curve shows that the accuracy to differentiate between patients with and without heart disease is the best in NT-proBNP compared to ANP levels.

In our patient group NT-proBNP had a positive predictive value (PPV) of 69% at a cutoff value of 200 ng l^−1^ and a negative predictive value (NPV) of 100%. No additional information was obtained by combining the NT-proBNP values with the ANP levels. To determine the accuracy of both diagnostic tests, a receiver operating characteristic (ROC) curve was used, which showed an area under the curve for NT-proBNP of 0.94 (95% CI 0.85–1.04) and for ANP of 0.69 (95% CI 0.44–0.96) (Figure 3

). The highest cutoff value of NT-proBNP with retaining a sensitivity of 100% was 300 ng l^−1^.

**Figure 4:**
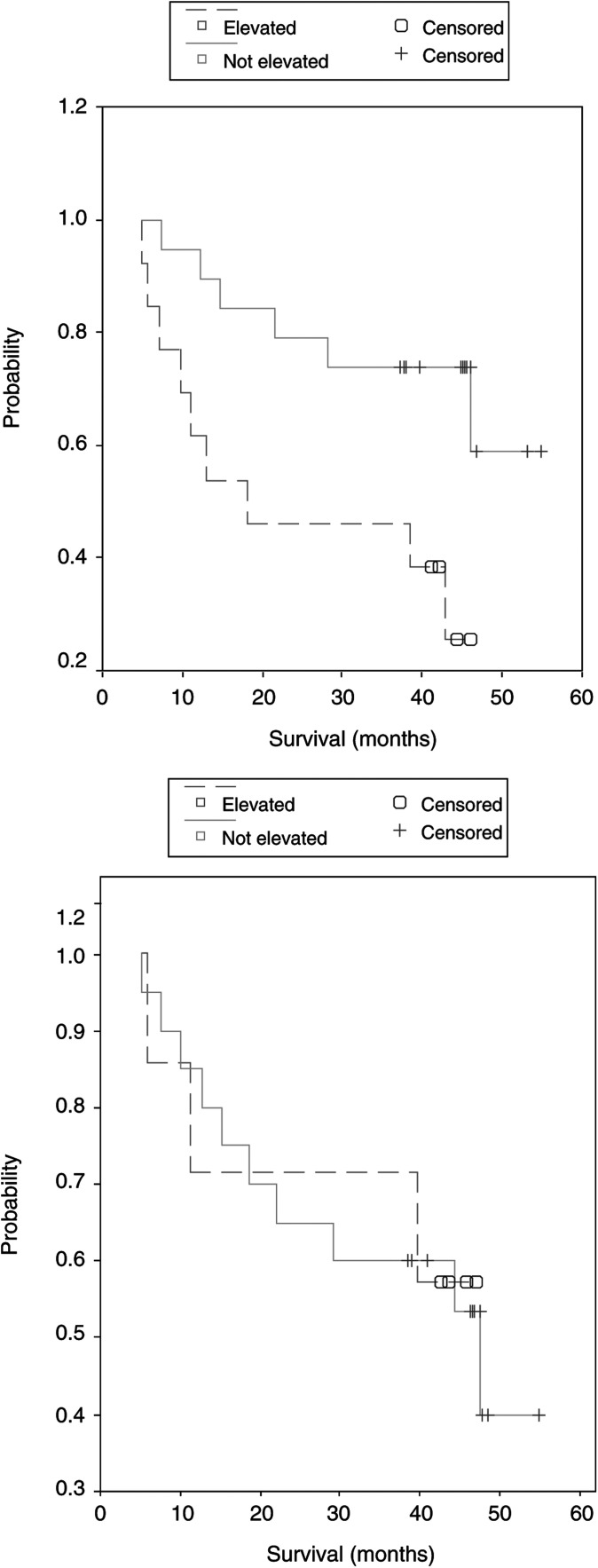
Kaplan–Meier curves show a significant better survival in patients with normal levels of NT-proBNP compared to those with elevated levels. This does not apply for the levels of ANP.

A significantly better survival was observed in patients with a normal NT-proBNP value compared to those with elevated levels (*P*=0.02). This difference was not seen in the group with a normal compared to an elevated ANP level (*P*=0.93) (Figure 4

).

## DISCUSSION

Thickening of the right heart valves caused by formation of fibrotic plaques eventually followed by regurgitation and right ventricular failure is a characteristic feature of CHD. In metastatic NET with production of hormones the development of CHD is reported in 20–70% of the patients (Tornebrandt *et al*, 1986; Robiolio *et al*, 1995; Westberg *et al*, 2001; Zuetenhorst *et al*, 2003) and in many patients attributed to the cause of death (Ross and Roberts, 1985). In the present series of 32 patients, the incidence of CHD is 28%, which is rather low compared to the results reported in literature. This might be due to the strict criteria we used for the definition CHD and the availability of octreotide the last decades has improved survival in these patients group with probably a less frequent development of CHD (Quaedvlieg *et al*, 2001).

In the follow-up and monitoring of carcinoid patients the echocardiography is the cornerstone in the diagnosis of CHD. However, performing an echocardiography is laborious, expensive and not always readily available as referral to a cardiologist is necessary. For these reasons, the cardiac evaluation of carcinoid patients without symptoms of heart failure is often performed less frequently than recommended. Clearly, a screening method allowing rapid and accurate differentiation between patients with and without CHD would be desirable. In this study with 32 patients, we found NT-proBNP to be a reliable marker to make this differentiation with a sensitivity of 100% and a specificity of 83%. This is comparable to the literature for diagnosis of cardiac dysfunction in the general population (McDonagh *et al*, 1998; Luchner *et al*, 2000) or in patients suspected to have heart failure (Cowie *et al*, 1997; Maisel *et al*, 2002,^2003^). The PPV of 69% as described in our study is relatively high compared to studies in the general population with a PPV of approximately 30% (Bay *et al*, 2003), but is in accordance with studies performed in a population with a higher chance of cardiac dysfunction (Cowie *et al*, 1997; Hammerer-Lercher *et al*, 2001; Maisel *et al*, 2002). In our carcinoid population, ANP was less reliable. An explanation could be the application of the activated ANP, which is less stable compared to the prohormone and NT-terminal fragment. However, earlier reports did show diagnostic values for activated ANP in carcinoid patients (Lundin *et al*, 1989; Zuetenhorst *et al*, 2003). Tested by a ROC curve, the diagnostic capacities of NT-proBNP were better compared to ANP, and no additional information was obtained by combining NT-proBNP with ANP. Similar to our findings, in earlier studies with a direct comparison between atrial and brain natriuretic peptides, an advantage for brain natriuretic peptides was convincingly proved with no increased predictive power by addition of ANP to BNP determination (Davidson *et al*, 1996; Cowie *et al*, 1997; McDonagh *et al*, 1998; Hammerer-Lercher *et al*, 2001).

Natriuretic peptides are mainly produced and excreted in the atria of the heart in response to increased wall tension. BNP, in contrast to ANP, is not only secreted from the atria, but also from the ventricles, especially in patients with heart failure. Moreover, there is a correlation between the degree of dilatation and levels of natriuretic peptides (Yasue *et al*, 1994). Similar to the literature, we also found a significant correlation between the levels of NT-proBNP and the degree of dilatation of the right atrium and ventricle. Although higher levels of ANP were detected in patients with severe dilatation of the right atrium and ventricle compared to those with only mild or no dilatation, this did not reach significance. Most studies about the influence of cardiac dilatation and levels of natriuretic peptides are performed in patients with left-sided heart failure. Information about natriuretic peptide excretion in right ventricular pressure overload, such as in CHD, is scarce and therefore comparison of our findings with other studies is difficult. In two studies of Tulevski *et al*, 2001a,2001b a relationship between levels of ANP and BNP with right ventricular dysfunction was reported. In our population, elevated levels of NT-proBNP were present in all patients with severe tricuspid valve regurgitation and a significant correlation between degree of regurgitation and NT-proBNP levels was found.

Several studies described the prognostic value of natriuretic peptides in patients with acute coronary syndromes and heart failure (Omland *et al*, 1996; de Lemos *et al*, 2001; Koglin *et al*, 2001; Richards *et al*, 2003). Patients with elevated levels of BNP were at a higher risk of dying, developing heart failure or undergoing a new myocardial event compared to those with normal levels. As might be expected, we also found a significant better survival for patients with normal levels of NT-proBNP compared to those with elevated levels.

In conclusion, NT-proBNP is a reliable marker to make a rapid and accurate differentiation between patients with and without CHD. Survival of patients with normal levels of NT-proBNP is better compared to those with elevated levels. As many patients with hormonal active NET die from cardiac causes, the detection of CHD in an early stage is important to adjust therapy and improve prognosis. A regular screening of NT-proBNP levels might direct the use of cardiac echography and guide treatment strategies.
